# Development of a digital tool for home‐based monitoring of skin disease for older adults

**DOI:** 10.1002/ski2.235

**Published:** 2023-06-28

**Authors:** Sven van Egmond, Zhuo R. Cai, Vanessa Nava, Isabella Joy de Vere Hunt, Bailie R. Rapaport, Justin Ko, Albert S. Chiou, Kavita Sarin, Jean Tang, Lucy Zhang, Eleni Linos

**Affiliations:** ^1^ Program for Clinical Research and Technology Stanford University Stanford California USA; ^2^ Department of Dermatology Stanford University School of Medicine Redwood City California USA

## Abstract

We developed a digital tool for home‐based monitoring of skin disease, our digital tool. In the current observational pilot study, we found that DORA is feasible to use in practice, as it has a high patient compliance, retention and satisfaction. Clinicans rated the photos generally good quality or perfect quality. These results show that the digital health tool DORA can easily be used by patients to send photos to their dermatologist, which could reduce unnecessary clinical visits. It may also be used in other settings where digital literacy barriers and unequal access to dermatologists contribute to healthcare disparities.

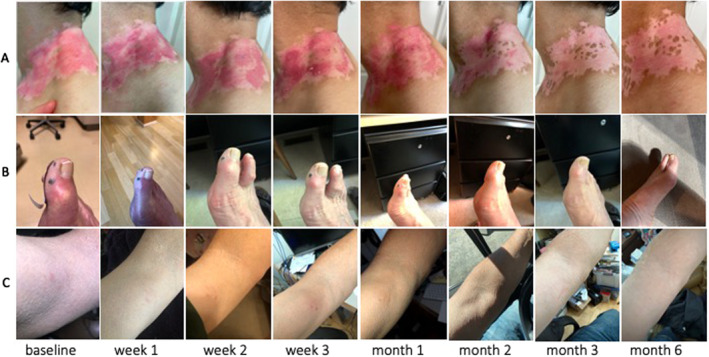

Dear Editor, The COVID‐19 pandemic has accelerated the adoption of teledermatology.^1^ However, current tools pose substantial barriers for older adults and those with low digital literacy.^2^, ^3^ By implementing user‐centred design principles, we developed a digital tool for home‐based monitoring of skin disease specifically designed for older adults. Our digital tool is a novel, text‐based virtual assistant, based on REDCap^4^ and Twilio (Twilio Inc) application programing interfaces. Our digital tool automates photo and self‐reported symptom collection, and allows communication between patients and the research team.

We evaluated the feasibility, usability, patient compliance, retention, and clinical utility of our digital tool. Eligible were patients ≥70 years, with any skin disease, access to a smartphone, who spoke English, and had no major cognitive impairment (<5 errors on the short portable mental status questionnaire).^4^ Patients were asked to send photos and answer a questionnaire about a solitary skin lesion initially weekly for 4 weeks, then monthly until 6 months, with a final request 9 months after baseline. This observational pilot study did not change patients' clinical care. We measured response time, photo quality, and participant satisfaction using the mHealth app usability questionnaire (MAUQ).^5^ MAUQ requests participants to assess 18 statements regarding three subscales of app usability (ease of use, information arrangement, and usefulness) on a seven point Likert scale with 1 as strongly disagree and 7 as strongly agree and was administered at week 8. Patients received $100 for their participation over the 9‐month period. This study was approved by the Stanford University Institutional Review Board (IRB #60677).

We recruited 62 patients from Stanford's Dermatology Clinic from August‐December 2021. Participants' mean age was 77 years (70–94), 39% female, 81% white, 18% high school or lower education, and 45% with a graduate or professional school degree. After a mean follow‐up time of 30 weeks, 76% (295/388) completed requests for photo submission (52% at initial request, 22% after first reminder and 26% after second reminder) and 72% (278/388) symptom questionnaires were completed. The median response time was 1.8 days (IQR 1.0–3.2). Four participants dropped out during the course of the study: one participant died from unrelated causes; one cited ‘lack of internet at new home’, and another cited ‘too busy’ as reasons for discontinuing. Median MAUQ scores were 5.6/7.0 (SD 1.3) for ease of use, 5.5/7.0 (SD 1.3) for interface satisfaction, and 5.2/7.0 (SD 1.3) for usefulness. Figure 1 shows examples of serial photos uploaded by three patients. Four dermatology clinicians evaluated the quality of the first 88 photos from 15 patients and rated 49% of photo series ‘generally good quality’ or ‘perfect quality’. Clinicians reported good confidence (scored 3.8/5.0, SD 1.1) in triaging skin diseases and moderate confidence in diagnosing and providing treatment recommendations based on the photos (3.0/5.0, SD 1.3 and 3.1/5.0, SD 1.3 respectively). To increase their confidence, physicians reported that photo quality should be improved; the lesion of interest in the photo should be indicated; and clinical information about the lesion and patient should be provided.

**FIGURE 1:**
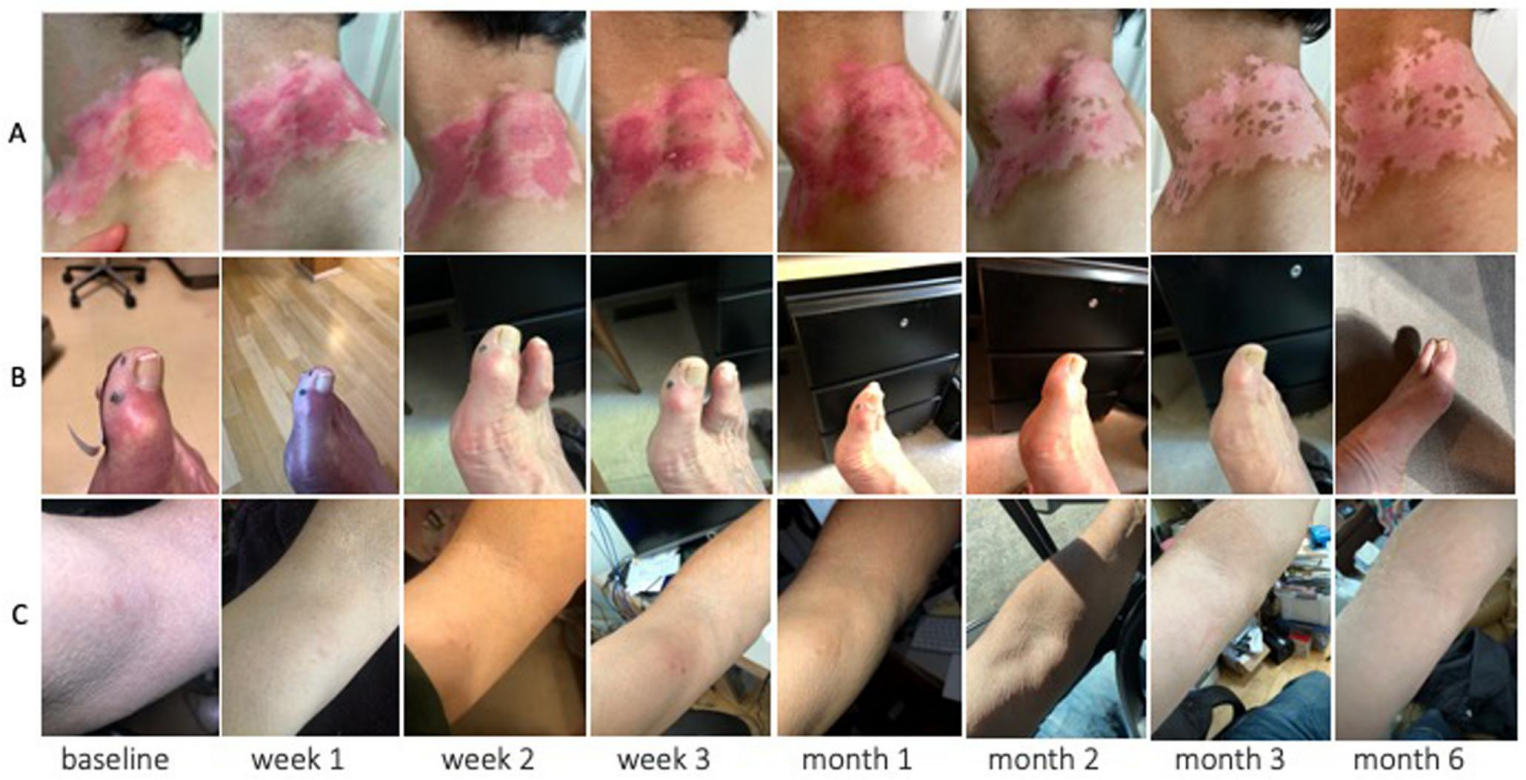
Examples of serial photos sent by participants using our digital tool. (a) Patient with vitiligo patch located on his neck receiving laser therapy. (b) Patient with blood blisters on his toe without treatment. (c) Patient known to have mycosis fungoides who had a suspicious lesion on his arm undergoing routine monitoring.

MHealth app usability questionnaire scores for our digital tool are comparable to other mobile healthcare apps, indicating good usability and satisfaction by older adults, and patient retention was high.^6^, ^7^ Clinicians were confident in making triage recommendations, but only moderately confident in diagnosing and making clinical decisions based on the photos. In order to increase physician confidence, clinically relevant information about the patient and lesion should be provided alongside the photo of the lesion. In addition, patients should be guided in the photo taking process to make sure the lesion is centred, in‐focus and well‐lit.

One limitation of this pilot study is that our participants were highly educated, with high levels of technological literacy, limiting the generalisability of our findings to broader populations. However, our explicit focus was to ensure usability for all older adults, with particular attention given to the needs of patients with lower levels of technological literacy. Our previous qualitative work indicates that teledermatology modalities which facilitate remote care can be particularly beneficial to older adults with transport or financial limitations to attending clinic in‐person; need for assistance from caregivers; or mobility issues.^1^ In the current feasibility pilot we monitored one skin lesion per participant. One of our next steps is to enable patients to send multiple photos of their skin through our digital tool.

In conclusion, this feasibility pilot demonstrates that the digital health tool Dermatology for OldeR Adults can easily be used by patients to send photos to their dermatologist. This could reduce unnecessary visits to the clinic, with reduction in travel time and associated burden notably pertinent to older adults with financial or mobility restrictions. Further, our digital tool requires further validation but has the potential to reduce healthcare disparities in other settings where digital literacy barriers to current teledermatology tools and unequal access to dermatologists present barriers to care.

## CONFLICT OF INTEREST STATEMENT

None to declare.

## AUTHOR CONTRIBUTIONS

**Sven van Egmond**: Data curation (Equal); Formal analysis (Equal); Methodology (Equal); Project administration (Lead); Resources (Equal); Writing – original draft (Lead); Writing – review & editing (Equal). **Zhuo R. Cai**: Data curation (Equal); Formal analysis (Equal); Methodology (Equal); Project administration (Lead); Resources (Equal); Software (Lead); Writing – original draft (Equal); Writing – review & editing (Equal). **Vanessa Nava**: Data curation (Equal); Project administration (Equal); Resources (Equal); Writing – review & editing (Equal). **Isabella Joy de Vere Hunt**: Formal analysis (Equal); Methodology (Equal); Writing – review & editing (Equal). **Bailie R. Rapaport**: Data curation (Equal); Project administration (Equal); Resources (Equal); Writing – review & editing (Equal). **Justin Ko**: Data curation (Equal); Project administration (Equal); Supervision (Equal); Writing – review & editing (Equal). **Albert S. Chiou**: Data curation (Equal); Project administration (Equal); Supervision (Equal); Writing – review & editing (Equal). **Kavita Sarin**: Data curation (Equal); Project administration (Equal); Supervision (Equal); Writing – review & editing (Equal). **Jean Tang**: Data curation (Equal); Project administration (Equal); Supervision (Equal); Writing – review & editing (Equal). **Lucy Zhang**: Conceptualisation (Equal); Methodology (Equal); Software (Equal); Visualisation (Equal); Writing – review & editing (Equal). **Eleni Linos**: Conceptualisation (Lead); Funding acquisition (Lead); Investigation (Lead); Methodology (Lead); Supervision (Lead); Writing – review & editing (Lead).

## FUNDING INFORMATION

Foundation for the National Institutes of Health, 1R21AG06698001, K24AR075060

## ETHICS STATEMENT

This study was approved by the Stanford University Institutional Review Board (IRB #60677).

## Data Availability

The data that support the findings of this study are available on request from the corresponding author. The data are not publicly available due to privacy or ethical restrictions.
